# An Ergonomic Assessment of Different Postures and Children Risk during Evacuations

**DOI:** 10.3390/ijerph182212029

**Published:** 2021-11-16

**Authors:** Xiaohu Jia, Bo Zhang, Xiaoyu Gao, Jiaxu Zhou

**Affiliations:** 1 Architecture College, Inner Mongolia University of Technology (IMUT), Hohhot 010051, China; gdjiaxiaohu@126.com (X.J.); zb1064389470@126.com (B.Z.); gaoxiaoyu0705@126.com (X.G.); 2 UCL Institute for Environmental Design and Engineering, The Bartlett, University College London (UCL), London WC1H 0NN, UK

**Keywords:** children risk assessment, postures, body joints, ergonomics, motion analysis

## Abstract

Crawling is recommended for avoiding high heat and toxic fumes and for obtaining more breathable air during evacuations. Few studies have evaluated the effects of crawling on physical joints and velocity, especially in children. Based on motion capture technology, this study proposes a novel method of using wearable sensors to collect exposure (e.g., mean duration, frequency) on children’s joints to objectively quantify the impacts of different locomotion methods on physical characteristics. An on-site experiment was conducted in a kindergarten with 28 children (13 boys and 15 girls) of different ages (4–6 years old) who traveled up to 22 m in three different postures: upright walking (UW), stoop walking (SW), and knee and hand crawling (KHC). The results showed that: (1) The level of joint fatigue for KHC was heavier than bipedal walking (*p* < 0.05), which was evidenced by higher mean duration and frequency. There was no significant difference between UW and SW (*p* > 0.05). (2) The physical characteristics of the children in the different postures observed in this study were different (*p* < 0.05). The ankle was more fatigued than other joints during bipedal walking. Unlike infants, the wrists and hips of the children became fatigued while crawling. The key actions flexion/extension are more likely to induce joint fatigue vs. other actions. (3) Crawling velocity was significantly slower than the bipedal velocities, and UW was 10.6% faster than SW (*p* < 0.05). The bipedal walking velocity started to decrease after the children had travelled up to 13 m, while the KHC velocity started to decrease after traveling up to 11.6 m. (4) In a severe fire, the adoption of SW is suggested, as the evacuees can both evacuate quickly and avoid overworking their joints. (5) There were no significant differences in the age (*p* > 0.05) and gender (*p* > 0.05) of the children on the joints in any of the three postures. To conclude, KHC causes more damage to body joints compared to bipedal walking, as evidenced by higher exposure (mean duration, frequency), whereas UW and SW are similar in terms of the level of joint fatigue. The above findings are expected to provide a useful reference for future applications in the children’s risk assessment and in the prevention design of buildings.

## 1. Introduction

Over the past decade, there have been an increasing number of articles in the ergonomics literature that have used wearable sensors to capture human motion data for analysis and decision-making. The relationship between the postures and the occupations of adults has been studied for a long time, and in terms of evacuation, researchers have found that adopting different postures significantly affects survival rates, but little is known about children. Moreover, children are more vulnerable than adults during evacuation [1,2,3,4], as they are physically, mentally, and physiologically underdeveloped. Recently, researchers have become increasingly interested in the motor skills and evacuation behavior of children [5,6,7,8]. However, ergonomic findings on the evacuation behavior of children with a motion capture system remain limited; therefore, an in-depth study concerning the dynamic behavior of children is necessary.

### 1.1. Evacuation Postures

During a fire, it is common for people to adopt different motor postures for evacuation. According to the National Fire Protection Association (NFPA 2015), the majority of deaths in a fire are not due to direct burns but are rather due to asphyxiation caused by the inhalation of toxic and hazardous smoke or high temperature heat. This is because as fire spreads through the building, it consumes most of the oxygen that is available in the building and produces toxic gases that fill the top parts of the floors in the building. As the fire grows further, smoke fills the space from the ceiling downwards. The toxic and hot smoke severely impairs people’s ability to escape and deteriorates the environmental conditions in the breathing zone. Therefore, the National Fire Protection Association recommends that evacuees choose stoop walking (SW) or knee and hand crawling (KHC) to obtain more oxygen during a severe fire, i.e., to avoid the hot and toxic smoke from the upper parts of the building space.

A limited number of studies in recent years have discussed the effects of different postures on evacuation. Cao et al. (2014) studied the effects of different postures on escape velocity [9]. Kady et al. (2009) explored the effects of person characteristics on crawling velocity [10]. Cao et al. (2018) previously investigated speed in five postures (two bipedal postures and three crawling postures) among university students. They demonstrated that crawling is much more difficult than walking, as evidenced by higher physiological indicators [11].

There are fewer existing studies on children. Najmanová et al. (2017) conducted group evacuation drills with young children and confirmed that age and the configuration of escape routes affect the self-rescue abilities of children [12]. Some researchers have collected other data on child evacuation, such as flow density, horizontal movement speed, vertical movement speed of children as well as on their choice of evacuation routes [2,13,14] Zhou et al. (2019) collected physiological data from children based on wearable sensors to quantitatively evaluate the effects of different types of alarm sounds on the perception of evacuation sound signals [15]. Wang et al. (2020) studied the KHC behavior of primary and secondary school students and showed that the adaptation time of secondary school students was lower than that of primary school students [16].

However, there are limited research findings on physical characteristics in different postures. Studies on the behavioral characteristics of evacuees in different evacuation postures using advanced equipment such as motion capture devices are especially rare.

### 1.2. Motion Capture System

In recent year, there has been an increase in research using motion capture sensors to collect relevant behavioral data due to emerging developments in technical equipment. However, research on children is sparse, particularly on their movements during evacuation. Cockcroftet et al. (2011) analysed movement data from 10 male cyclists on the road using a motion capture system. However, the results demonstrated that the data collected near the pedal and handlebar interface were not acceptable due to magnetic interference from the system [17]. With the further development of wearable sensor technology, the magnetic interference problem has been solved, and human motion information can be collected with high accuracy, even in open environments [18,19,20]. Long Liu et al. (2021) took a systematic approach based on multi-sensor fusion technology to evaluate the training performance of kayakers [21]. B. Wu et al. (2020) collected information from elderly Japanese lawn mowers mowing on an inclined surface using a highly sophisticated motion capture device to compare the hazards of mowing movements under three different working conditions [22]. In addition, some researchers have used motion capture systems to study the gait of children in normal situations (non-emergency situations) [23,24,25,26,27,28,29].

A number of experimental methods are not applicable to children compared to adults. For example, some studies have used self-reporting and interview methods to examine the behavior of children. Mytton, J et al. (2017) explored children’s accounts of their experiences in accidental house fires to inform the UK Fire and Rescue Service in order to provide training in rescue services and in setting up relevant fire safety education courses [24]. Moreover, these are not objective and do not allow for the collection of real-time and realistic data. Some researchers used virtual reality to study evacuation behavior [25,26], but this method requires detailed instructions being given to children. Participants should be fully familiar with the process before starting the task, which is already too complex for children and is therefore not applicable to them.

Overall, research on children in different postures during evacuation remain limited. We still do not know the relationship between the risks to the safety of a child’s joints that are associated with different postures. Therefore, there is an increasing need for an objective method to quantitatively assess the effects of different evacuation postures on children’s joints and their evacuation speed.

### 1.3. Aims and Contributions

In previous literature, few studies have examined the mechanisms of postural effects on their risk to children. In order to fill this gap, this paper analyses the ergonomic effects of different postures and their risk to children during evacuations. An accelerometer-based motion capture device with 14 sensors was used to collect the data. The physical characteristics of the children in each posture and the variability between the different postures were analyzed using two indicators (mean duration and frequency). This paper focused on the following areas:

What are the features of children’s joints in different postures (UW, SW, KHC)? What are the effects of different postures on the ability of children to evacuate safely?

Does age/gender have an effect on the joints in different postures?

## 2. Methodology

**Figure 1:**
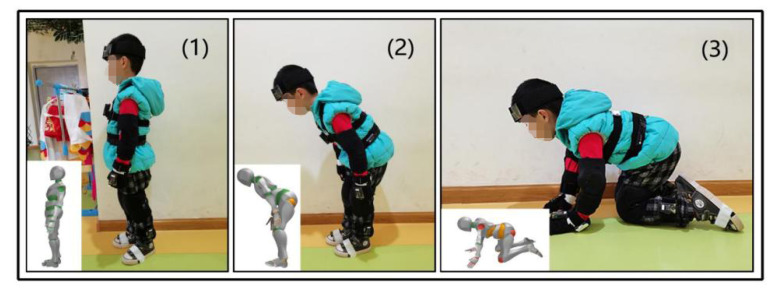
Evacuation postures. (1) upright walking; (2) stoop walking; (3) knee and hand crawling.

In this study, the kinematic joint angles of different evacuation postures were detected by wearable motion capture sensors. Each subject participated in three separate trials (travel up to 22 m) using five different postures, including (1) upright walking (UW), (2) stoop walking (UW), and (3) knee and hand crawling (KHC) (Figure 1). The motion capture device detected joint exposure in three postures. The whole experiment was recorded using a camera mounted on the trolley. The sensors obtained the raw data, and the motion capture system processed the raw data to obtain the exposure of each joint. Finally, IBM SPSS Statistics 25.0 analyzed the relationship between the exposure of each joint and its confidence level.

### 2.1. Participants

According to Tatuic and Dederich’s [27] study on the ability of preschool children to protect themselves, the minimum age for preschool children at which children can understand and can carry out simple instructions is 2.5–3 years old. Therefore, the subjects of this experiment were 28 children (13 boys and 15 girls) aged 4–6 years from a senior class, who were older than 3 years old. The inclusion criteria are listed below. The children studied at Hua Di Kindergarten, and they were in good health. Most importantly, they did not manifest any COVID-19 symptoms. None of them had any physical impairments or disabilities. The children’s body data measurements were taken at the preschool, two hours after the children entered school (after breakfast). A TANITA WB-3000 device was used to record height, weight, and BMI. Physical data of the participants are shown in Table 1. This study was approved by the Hua Di Kindergarten and the children’s parents, and written informed consent was signed. Ethical approval for this study was also provided by the Ethics Committee of the College of Architecture, Inner Mongolia University of Technology.

### 2.2. Experimental Setting and Equipment

A medium-sized kindergarten called Hua Di Kindergarten (in Hohhot, China) was selected as the experimental site, as it met the common features of a preschool building in China [13,15,28]. Hua Di Kindergarten, a three-story building, provides three different age-level classes: senior class (5–6 years), medium class (4–5 years), and junior class (3–4 years). The experiment was conducted in the third-floor corridor, as it is relatively quiet on the top floor. This was to reduce any interferences from other people during the experiment.

**Figure 2:**
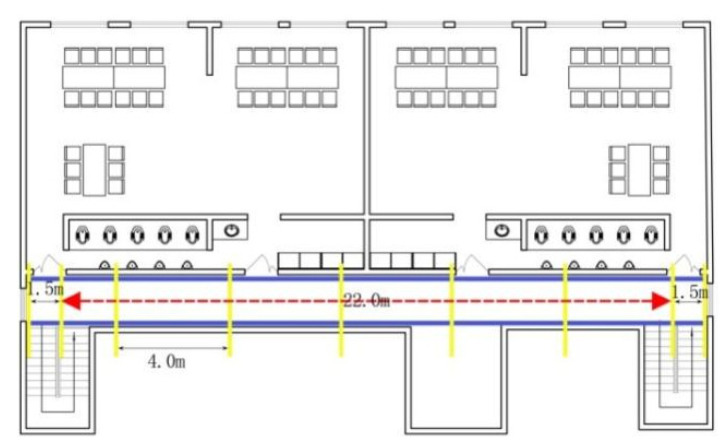
Test track.

According to the International Code of Construction (ICC) (2018) standard requirements, kindergartens with automatic sprinkler systems should have an exit distance of no more than 22.86 m) [29]. The test track was 22 m in length (this satisfies the Chinese building design codes [30]) and was divided into six segments to detect potential velocity changes. There is an original layer of plastic flooring on top of the concrete, which is a common indoor floor covering material for kindergartens in China. The start and finish lines were set 1.5 m from the beginning and the end of the track to control any acceleration and deceleration effects (Figure 2).

**Figure 3:**
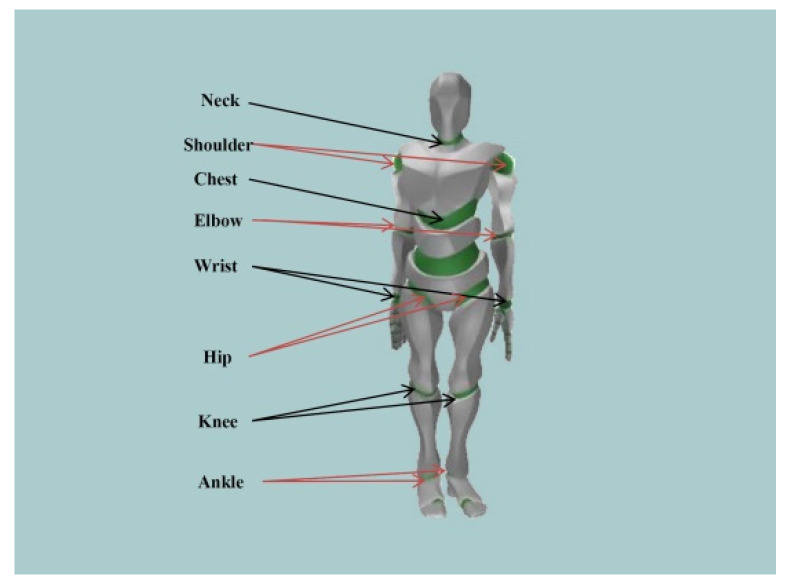
Representation of experiments.

**Figure 4:**
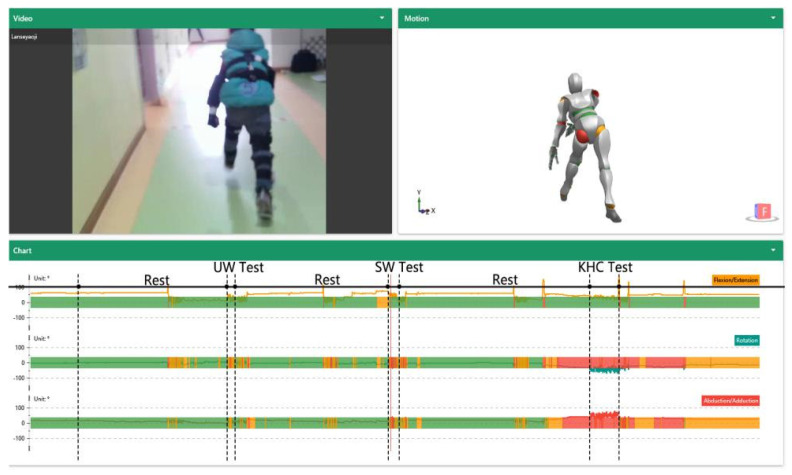
Interface of the experiment data collection platform. UW—upright walking; SW—stoop walking; KHC—knee and hand crawling.

A captive accelerometer-based motion capture device (*CAPTIVE Motion*, USA) with 14 wearable sensors was used to assess the children’s behaviors. It collected the 3D coordinate information of the subject’s joints with a frequency of 64 HZ [31,32,33,34,35,36]. The motion sensors integrated a 3-axis accelerometer, a 3-axis gyroscope, and a 3-axis magnetometer with powerful fusion algorithms. Then, the angles, angular speed, and angular accelerations were converted in ErgoLAB software. A total of 14 motion capture sensors were placed on the head, chest, left upper arm, left forearm, left hand, right upper arm, right forearm, right hand, left upper leg, left foreleg, left foot, right upper leg, right foreleg, and right foot to detect the following joints: neck, chest, left shoulder, left elbow, left wrist, right shoulder, right elbow, right wrist joint, left hip, left knee, left ankle, right hip, right knee, and right ankle (Figure 3). The motion direction and rotation of the limb joints in 3D space were calculated by the motion capture system, which had high robustness and fast tracking. The computer displayed the subject’s movements in real-time and recorded the data information for each joint [37,38]; the interface of the experiment data collection platform is shown in Figure 4.

### 2.3. Experimental Procedure

The three postures were randomly assigned to the children to negate potential order effects (i.e., learning and fatigue). Due to the specific nature of the subject population (children need a midday break), the duration of the experiment (including wearing the sensors, the experiment, and breaks) had to be limited to two hours per subject. The final experiment was conducted from 10:00 am to 11:30 am and from 3:00 pm to 4:30 pm each day. Each subject was required to rest for at least 2 h prior to the experiment to ensure that they were well hydrated and caffeine-free.

Before the experiment, each subject’s age, gender, height, and weight were recorded, and each subject was labelled (blue labels for boys and red labels for girls) and numbered in order. The researcher instructed the subjects about the evacuation posture. Before putting on the sensors, the 14 wearable motion capture sensors were put on the ground for ten minutes, and then each sensor was switched on. The signal light turning to green indicated that the sensor was on, at which point the computer could search for each sensor’s frequency band signal. The sensors were attached to the appropriate part of the joints with straps. Meanwhile, the researcher clicked the initialize button on the computer-based motion capture system, and the subjects sat down in a chair to wait to begin.

The start sign of the experiment was marked by the subject’s entire body passing completely over the starting line after hearing the command.

During the experiment, each subject was required to perform the experiment in three different postures: (1) upright walking (UW); (2) stoop walking (SW); and (3) knee and hand crawling (KHC). Subjects were required to wear knee pads, elbow pads, and gloves during KHC. The subjects were instructed to complete the trials as rapidly as possible while maintaining the tested posture. At the same time, an investigator closely followed each subject while pushing a trolley with a camera and a computer to record the entire experiment, and the recorded video was used to determine the intermediate times and velocities as the subjects passed over each track section. After completing the experiment in each posture, subjects were provided sufficient rest between successive trials to reduce the effects of tail retention and to avoid confounding effects caused by fatigue. The subjects had the right to rest or stop the experiment at any time during each experiment.

A stop sign was marked by any physical discomfort experienced by the subject; a malfunction of the test equipment; and a request from the subject to terminate [11,39]. The reference for passing time was when each subject’s entire body passed the line of each segment and finish line.

### 2.4. Data Analysis

During the experiment, one girl stopped the experiment due to physical exhaustion during the crawling experiment, so the final data was collected for the complete experiment with 27 subjects (13 boys and 14 girls). A database containing the final results was created using IBM SPSS 25.0 (IBM, New York, NY, USA) to evaluate the effects of different postures on the risk level of the children’s joints.

Based on the manual for the motion capture device, which is able to record the 3D coordinates of the joints every 4 milliseconds (ms), the motion capture device can record the 3D coordinate from a total of 14 types of joint. Different joints contain different key actions, with the chest and neck containing three movements: lateral flexion (LF), flexion/extension (FLE/EXT), and rotation (ROT), and with the ankle and elbow containing two actions: flexion/extension (FLE/EXT) and rotation (ROT). The hip contains three actions: adduction/abduction (ADD/ABD), flexion/extension (FLE/EXT), and rotation (ROT). The knee and the wrist contain two actions: adduction/abduction (ADD/ABD) and flexion/extension (FLE/EXT). There are three actions in the shoulder: horizontal rotation (HR), vertical rotation (VR), and rotation (ROT). The motion capture sensor has a built-in accelerometer sensor, gyroscope, etc., and data fusion processing that is conducted by an extended Kalman filter to calculate the angle of the joint from the subject’s 3D coordinate data [36,40].

According to the angle calculation formula [41], the angle of the joint can be calculated using the joint coordinates. Taking the ankle as an example, assuming that the subject’s initialized 3D coordinates are A (X_a_, Y_a_, Z_a_) for the left foot, B (X_b_, Y_b_, Z_b_) for the left ankle, and C (X_c_, Y_c_, Z_c_) for the left knee, two vectors $\vec{\mathrm{BA}}$, $\vec{\;\mathrm{BC}}$ were calculated first:

(1) $$\vec{BA}=(X_{a}\;-X_{b},\;Y_{a}-Y_{b},\;Z_{a}-Z_{b})$$

(2) $$\vec{\;BC}=(X_{c}-X_{b},\;Y_{c}-Y_{b},\;Z_{c}-Z_{b})$$

Then, the angle ***θ_Ankle_*** will be:

(3) $$\theta_{\mathrm{Ankle}}=\arccos\;\frac{\;\vec{BA}\times \vec{\;BC}}{\left|\vec{\;BA}\;\right|\times \left|\vec{\;BC\;}\right|}$$

Following the same calculation, other joints can be obtained. Excluding the angles for the chest and neck, the data for the other joints were collected in pairs (e.g., right and left ankle) [20].

**Figure 5:**
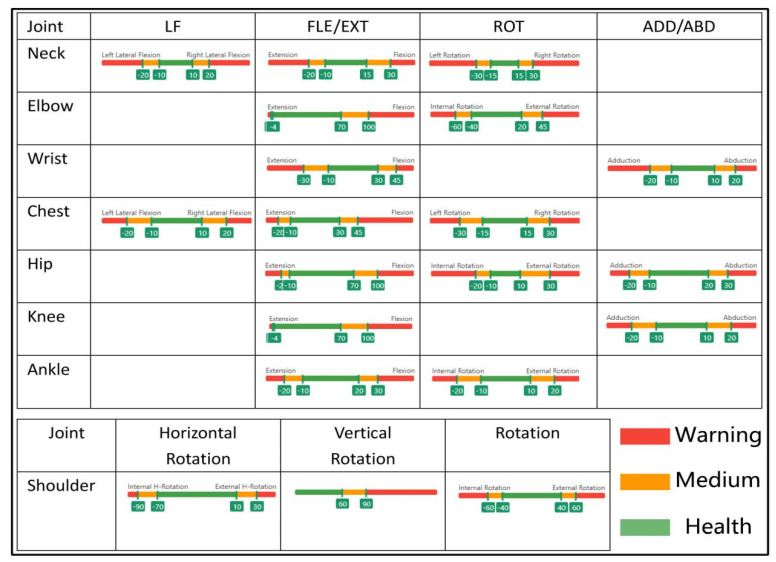
The threshold of joints. LF—lateral flexion; FLE—flexion; EXT—extension; ROT—rotation; ADD—adduction; ABD—abduction.

The risk level of each action was determined according to the joint threshold shown in Figure 5 [32,42,43,44,45,46,47]. Three colours were used to represent three risk levels to a certain extent: red meant that the joint was in an alert state, orange meant that the joint was in a moderate state, and green meant that the joint was in a healthy state. In terms of joint exposure, the software analyses the mean duration (S) and frequency (N/MIN) for the three colours for each joint. Joints are prone to fatigue or can be injured if the joint is on alert for a long time.

A database was created, and the data were statistically analysed for the physical characteristics in the three different postures. The independent variables included the three postures, gender, and joints. The dependent variables were the mean duration (S) and frequency (N/MIN) of the red, orange, and green colours, and the joint exposure was quantified using IBM SPSS 25.0 [48]. Finally, the related variable measurements were compared among these three postures and joints using an analysis of variance (ANOVA). Based on paired *t*-tests, the related variable measurement was to evaluate the differences between the exposure data corresponding to gender. The level of variability between the data was tested by repeated measurements and was based on a 95% confidence level.

## 3. Results

### 3.1. Evacuation Postures

#### 3.1.1. Upright Walking

**Figure 6:**
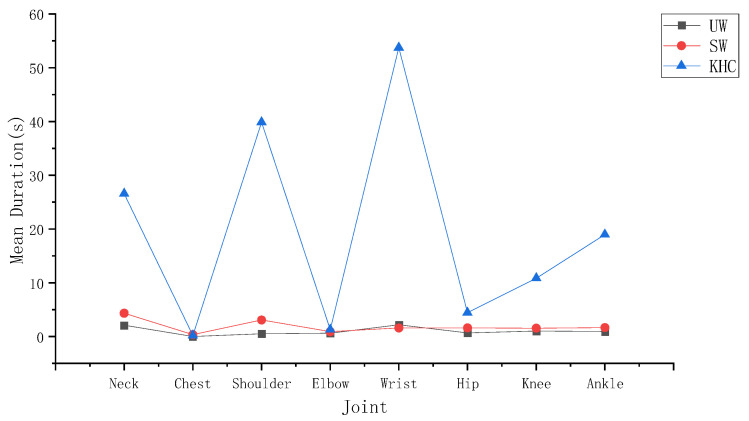
The mean joint duration. UW—upright walking; SW—stoop walking; KHC—knee and hand crawling.

**Figure 7:**
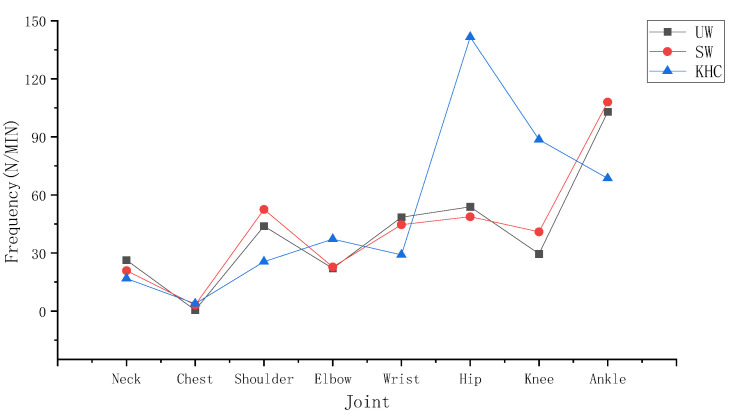
The joint frequency. UW—upright walking; SW—stoop walking; KHC—knee and hand crawling.

During upright walking, several characteristics can be observed according to Figure 6 and Figure 7. First, the wrist had the highest mean duration of angles above the alert threshold (2.34 S) among the joints measured in UW. The ankle had the highest frequency of angles above the alert threshold (105.89 N/MIN) among the joints. Second, to further analyse the level of variability between the joints, we conducted an ANOVA test, which showed that there was a significant difference both between the neck and other joints (*p* < 0.05) and between the ankle and other joints (*p* < 0.05). In conclusion, the neck and the ankle are more prone to fatigue than the other joints during UW.

To further analyse which key action makes the wrist and ankle more fatigued in UW, we conducted a paired *t*-test to determine the differences between the exposure corresponding to different actions. According to Table 2, in terms of the mean duration of the wrist, the key action ADD/ABD (1.5 S) was close to the FLE/EXT (1.29 S). the results indicated that no significant difference was observed for the wrist measurements (*p* > 0.05) in UW. Similarly, in terms of the ankle frequency, the key action FLE/EXT (83.55 N/MIIN) was 2.36 times higher than the ROT (21.887 N/MIN). The ankle measurement (*p* < 0.05) was significantly different between FLE/EXT and ROT, which means that FLE/EXT was more likely to fatigue the ankle than ROT during UW.

#### 3.1.2. Stoop Walking

In stoop walking, as seen in Figure 6 and Figure 7, the neck had the highest mean angle duration exceeding the alert threshold (4.58 S) among the joints measured during SW. The ankle had the highest angle frequency exceeding the alert threshold (110.47 N/MIN) among the joints measured in SW. To further analyse the level of variability between the joints, we conducted an ANOVA test, which showed significant differences both between the neck and the other joints (*p* < 0.05) and between the ankle and the other joints (*p* < 0.05). In conclusion, the neck and ankle are more prone to fatigue than other joints during SW.

To further demonstrate which key action made the neck and ankle more fatigued during SW, we conducted paired *t*-tests for the ankle and ANOVA tests for the neck. According to Table 2, in terms of the mean duration of the neck, the key action FLE/EXT (4.14 S) was much longer than the ROT (0.22 S) and the LF (0.22 S). Moreover, according to Table 2, the neck measurements during SW (*p* < 0.05) were significantly different among the key actions FLE/EXT, ROT, and LF, which means that FLE/EXT was more likely to fatigue the neck than ROT and LF. In terms of the ankle frequency, the key action FLE/EXT (85.13 N/MIIN) was 1.69 times higher than ROT (25.34 N/MIN). The ankle measurement (*p* < 0.05) was significantly different between FLE/EXT and ROT, which means that FLE/EXT was more likely to fatigue the ankle than ROT during SW.

#### 3.1.3. Knee and Hand Crawling

During knee and hand crawling, as seen in Figure 6 and Figure 7, the wrist had the highest mean duration angle exceeding the alert threshold (88.26 S) among the joints measured during KHC; the hip had the highest angle frequency exceeding the alert threshold (142.75 N/MIN) among the joints. To further analyse the level of variability in each joint, we conducted an ANOVA test, which showed significant differences both between the wrist and the other joints (*p* < 0.05) and between the hip and the other joints (*p* < 0.05). In summary, the wrist and hip are more prone to fatigue than the other joints during KHC.

To further analyse which key action makes the wrist and hip more fatigued in KHC, we conducted data statistics with paired *t*-tests for the wrist and ANOVA tests for the hip. In terms of the mean duration of the wrist, the key action FLE/EXT (59.70 S) was 109% higher than that of ADD/ABD (28.56 S). As shown in Table 2, there was a significant difference between the action FLE/EXT and ADD/ABD (*p* < 0.05), which means that FLE/EXT was more likely to fatigue than the wrist than ADD/ABD. In terms of the frequency of the hip, the key action ROT (56.85 N/MIIN) was 41% and 25% higher than ADD/ABD (40.25 N/MIN) and FLE/EXT (45.65 N/MIN), respectively. According to Table 2, however, the results indicate that no significant difference was observed for the measurement of the hip (*p* > 0.05) in KHC.

#### 3.1.4. Comparison of the Three Situations

We evaluated the effect of different postures on the joints based on the mean duration and the frequency of joints reaching the alert thresholds in different postures. To further validate the results, the related variable measurements were compared among these three situations using the ANOVA test, and all were tested at a 95% confidence level.

**Figure 8:**
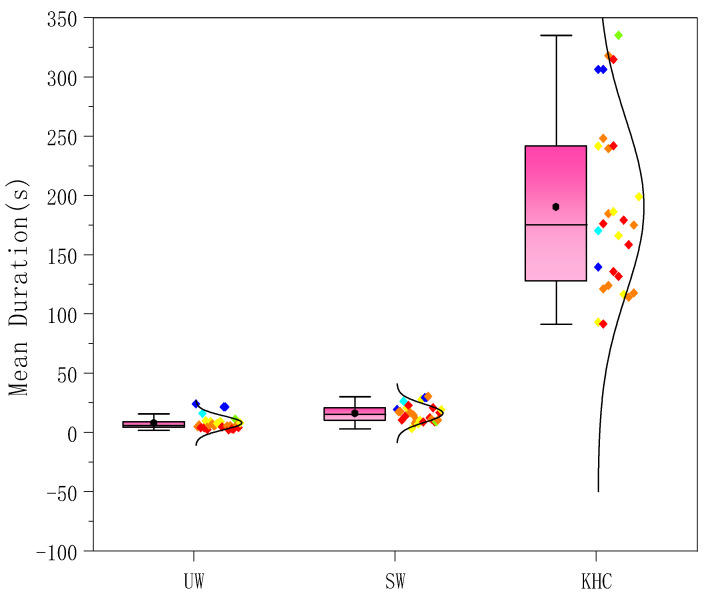
The mean duration of joints in three postures. UW—upright walking; SW—stoop walking; KHC—knee and hand crawling.

The mean duration of each joint in the different postures was counted for the subjects. Figure 8 shows the average for the 27 subjects. The mean duration of the joints during KHC far exceeded that of UW and SW. According to Table 3, there were significant differences among the three postures (*p* < 0.05), specifically between KHC and UW (*p* < 0.05) and between KHC and SW (*p* < 0.05).

**Figure 9:**
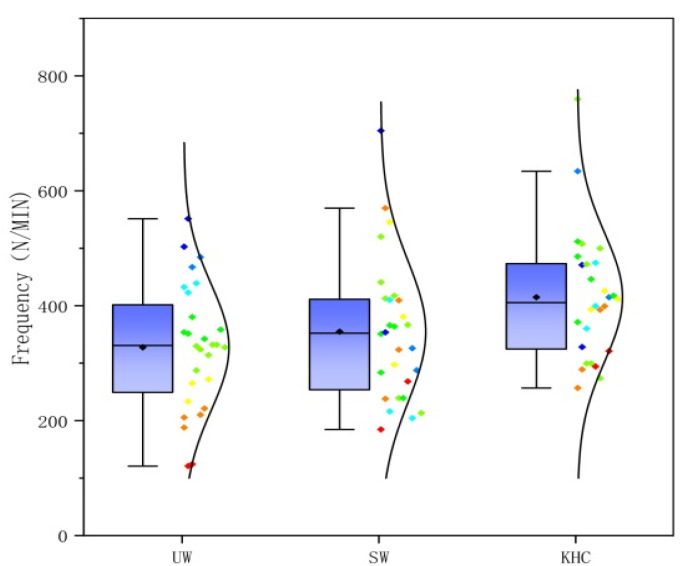
The frequency of joints in three postures. UW—upright walking; SW—stoop walking; KHC—knee and hand crawling.

The frequency of each joint in different postures was counted for the subjects. Figure 9 shows the average for the 27 subjects. The relationship for the frequency among the three postures was KHC > UW > SW, with KHC being 26.57% and 16.88% higher than UW and SW, respectively. As seen in Table 3, there was a significant difference (*p* < 0.05) among the three postures, specifically between KHC and SW (*p* < 0.05) and between KHC and UW (*p* < 0.05).

### 3.2. The Effect of Age/Gender on Children’s Joints

In order to analyse whether gender/age had an effect on the joints when the children were in different postures, statistical analyses were conducted, and the data were subjected to paired *t*-tests for gender and ANOVA tests for age.

#### 3.2.1. Gender

To analyse whether there was an effect resulting from gender on the joints in different postures, independent *t*-tests were conducted, and a 95% confidence level test was performed. As shown in Table 4, although the mean duration and frequency in the three postures were different, the independent *t*-tests indicated that the mean duration and frequency were not significantly different in three different postures for the gender measurement (*p* > 0.05).

#### 3.2.2. Age

To analyse whether there was an effect of age on the joints in different postures, we conducted an ANOVAL test. In the experiment, we collected the complete motion data from 27 children, 8 of whom were four years of age, 9 of whom were five years of age, and 10 of whom were six years of age.

As shown in Table 5, although the mean duration and frequency in three postures were different, the ANOVA tests indicated that the mean duration and frequency were not significantly different in the three different postures for the age measurement (*p* > 0.05).

**Figure 10:**
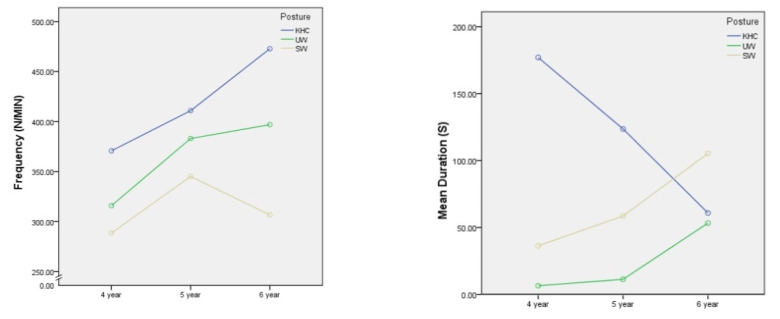
The estimated marginal mean of joints exceeding the threshold. UW—upright walking; SW—stoop walking; KHC—knee and hand crawling.

It is worth noting that when analysing the relationship between motion indicators and age, an ANOVA test was conducted. As shown in Figure 10, in the KHC posture, there is a decreasing trend in the estimated marginal means of the mean duration of joint as age increases, but the frequency increases. One possible reason for this is that children become more aware of their bodies and their ability to control them as they grow older. During KHC, when the joint angle exceeds the alert threshold, older children may immediately perceive the discomfort and make appropriate adjustments to continue crawling, whereas younger children may not be able to perceive the discomfort immediately or have no ability to adjust in time because they are not as well developed as older children. This results in a longer mean duration in younger children, while older children make appropriate adjustments resulting in an increase in frequency and a decrease in mean duration.

## 4. Discussion and Limitation

This study provides a new method to assess the effects of different evacuation postures on children’s body joints. Based on a field experimental analysis of three postures, upright walking (UW), stoop walking (SW), knee and hand crawling (KHC), this study presents the potential of motion data collected from wearable motion capture sensors to understand children’s joints during evacuation by showing differences in the mean duration and the frequency among different types of postures and the effects of gender and age.

The result of the level of joint fatigue for knee and hand crawling was heavier than bipedal walking, which is similar to the results of previous studies of evacuation postures in adults [11]. This study showed that the wrist (*p* < 0.05) and hip (*p* < 0.05) were more fatigued in KHC. The results are similar to the results of previous studies on adults [30,49]. However, our results different from those obtained during a study on infants when they crawl (Li Zhang et al., 2019). The level of distal limb joint (wrist and ankle) fatigue was heavier than proximal joint (shoulder and hip) fatigue [50]. The possible reason for this could be due to the different growth and development of body at different stages.

In addition, we collected the evacuation speed of the subjects in different postures. The results showed that different postures were affected significantly (*p* < 0.05).The average UW speed was measured at 2.29 ± 0.53 m/s, the average SW travel speed was measured at 2.07 ± 0.62 m/s, and the average KHC travel speed was measured at 0.58 ± 0.20 m/s. The average bipedal walking velocity measured during this study was faster than the velocity of children measured in previous studies [12,21,22]. This study focused on individual behavior rather than group behavior, which would be less likely to collide. As uch the results may be faster than previous studies. Moreover, the children were even faster than adults are in bipedal walking [10,11]. Similar results could be found in previous child evacuation studies [21]. This new finding indicates that children are slower than adults during KHC. A paired *t*-test showed that bipedal walking was significantly faster than crawling (*p* < 0.05). UW was faster than SW (*p* < 0.05). The velocity relationship among the different postures is similar to that of previous studies [11]. Quadratic fitting was conducted to observe the relationship between travel distance and individual velocity. For average velocity, the adjusted R^2^ of over 0.8 were achieved, with the exception of the KHC. For the fitted situation, the highest speed was found at the distances of 13.08 m, 13.17 m, and 11.60 m under UW, SW, and KHC posture, respectively. The results are different from the study on adults, whose velocities for KHC decreased significantly after traveling up to 9.14 m [11].

Our findings suggested that SW is recommended when there is a large amount of smoke. The respirable height is reduced based on the comparison analysis relationship among the postures, joints, and travel velocities. As the speed of SW, which is larger than KHC, is similar to the UW, the joint fatigue level of SW is not significantly different from UW but is lower than KHC. When adopting KHC, it is necessary to consider the effects of the reduced evacuation speed on the evacuation time. The negative impact of crawling on the joints is the heaviest among the three situations, and crawling for a long time will cause joints fatigue or could even lead to injury. It is also important to note that, in addition to the 22 m distance covered in the experiment, the children would have to continue walking during an actual evacuation. The travel velocity is likely to be further reduced due to the accumulation the fatigue level in the joints, ultimately affecting the risk level of the children, especially during emergency situations.

Although this study successfully demonstrates the use of motion data obtained from a motion capture device to study the physical characteristics of children in different evacuation postures, the study still has some drawbacks. Firstly, the study was conducted in a controlled evacuation environment (non-emergency situations). In actual evacuation scenarios, the data may be affected by factors such as obstacles, limited visibility, and varying building geometries. Secondly, this study only used 14 motion capture sensors to monitor the movement metrics of 14 joints and did not consider the effects of other joints such as the fingers and toes. Thirdly, the physical characteristics of the children in each posture and the variability between the different postures were only analysed by two kinematic indicators (mean duration and frequency). There is no information the kinetic joint parameters (joint torques, power output) and muscle activity for the three evacuation postures, which will likely be a direction for future research. Finally, only 28 subjects were involved in this study. A large cohort study should be studied in the future to understand risk and occupant behaviors.

## 5. Conclusions

This study proposes a novel method to evaluate the effect of different evacuation postures on the joints and velocity of children through an accelerometer-based motion capture device with 14 sensors. In this study, we collected a total of 14,580 items of motion data from 28 children (13 boys and 15 girls) aged 4–6 years old to calculate the exposure of the children’s joints. The physical characteristics of children in each posture and the variability between different postures were analysed by two indicators (mean duration and frequency). Through data analysis, the following conclusion may be drawn:

First of all, the level of joint fatigue for knee and hand crawling was heavier than bipedal walking, as evidenced by the higher mean duration and frequency. No significant difference between upright walking and stoop walking was suggested. Second, the ankle was more fatigue than other joints in bipedal walking, while the wrist and hip were more fatigue while crawling. The key action (flexion/extension) are more likely to induce joint fatigue compared to other actions. Furthermore, the crawling velocity was significantly slower than the bipedal walking velocities, and the upright walking velocity was faster than the stoop walking velocity. The bipedal walking velocity starts to decrease after traveling up to 13 m, while the knee and hand crawling velocity starts to decrease after traveling up to 11.6 m. No significant differences in age and gender were observed on the joints in three different types of postures.

## Figures and Tables

**Table 1:** Details of Participants in the Experiment.

Gender	Number	Age (Years)		Height (cm)		Weight (kg)		BMI (kg/m^2^)	
		Mean	SD	Mean	SD	Mean	SD	Mean	SD
Boys	13	5.0	0.82	116.31	6.84	22.07	4.48	16.17	1.71
Girls	15	5.4	0.91	116.20	5.85	20.75	2.93	15.34	1.42

Abbreviation: BMI, body mass index.

**Table 2:** Kinematic characteristics of joints in different postures.

Posture	Joint	Action	Mean	*p*
UW	Wrist	ADD/ABD	1.50	0.40
		FLE/EXT	1.29	
	Ankle	ROT	21.87	**0.00**
		FLE/EXT	83.55	
SW	Neck	FLE/EXT	4.14	**0.00**
		ROT	0.22	
		LF	0.22	
	Ankle	ROT	25.34	**0.00**
		FLE/EXT	85.13	
KHC	Wrist	FLE/EXT	59.70	**0.00**
		ADD/ABD	28.56	
	Hip	ADD/ABD	40.25	0.18
		FLE/EXT	45.65	
		ROT	56.85	

UW—upright walking; SW—stoop walking; KHC—knee and hand crawling; LF—lateral flexion; FLE—flexion; EXT –extension; ADD—adduction; ABD—abduction; ROT—rotation. Paired *t*-tests (for wrist, ankle) and ANOVA tests (for neck, hip) performed between motion indicators. The significant difference has been bolded.

**Table 3:** Relationship among postures with motion-capture measures.

Motion Indicators	UW-SW	UW-KHC	SW-KHC	KHC-SW-UW
	*p*	*p*	*p*	*p*
Mean Duration	0.484	**0.000**	**0.000**	**0.000**
Frequency	0.378	**0.006**	0.054	**0.018**

UW—upright walking; SW—stoop walking; KHC—knee and hand crawling. Paired *t*-tests peformed between postures on motion indicators. The significant difference has been bolded.

**Table 4:** Relationship between gender with motion-capture measures.

Motion Indicators	Gender	UW		SW		KHC	
		Mean	*p*	Mean	*p*	Mean	*p*
Mean Duration (S)	Boy	9.58	0.637	41.01	0.689	138.01	0.146
	Girl	8.39		31.38		194.70	
Frequency (N/MIN)	Boy	382.04	0.157	384.83	0.065	406.65	0.648
	Girl	317.13		298.67		429.99	

UW—upright walking; SW—stoop walking; KHC—knee and hand crawling. Independent *t*-tests performed between gender on motion indicators.

**Table 5:** Relationship among ages with motion-capture measures.

Behavioral Indicators	Age	UW		SW		KHC	
		Mean	*p*	Mean	*p*	Mean	*p*
Mean Duration (S)	4	6.47	0.388	36.32	0.610	176.98	0.125
	5	11.27		58.57		123.58	
	6	8.71		18.09		60.84	
Frequency (N/MIN)	4	315.89	0.673	288.50	0.429	370.72	0.119
	5	383.03		345.10		410.89	
	6	348.28		369.21		472.81	

UW—upright walking; SW—stoop walking; KHC—knee and hand crawling. ANOVA tests performed among ages on motion indicators.
